# Effects of a theory driven and culturally tailored educational program on promoting cervical cancer screening in rural populations

**DOI:** 10.1038/s41598-025-02600-z

**Published:** 2025-05-27

**Authors:** Mengyue Zhang, Janet W. H. Sit, Kai Chow Choi, Ka Ming Chow, Carmen W. H. Chan

**Affiliations:** The Nethersole School of Nursing, Faculty of Medicine, The Chinese University of Hong Kong, Hong Kong, China

**Keywords:** Cervical cancer, Cancer screening, Health education, Rural health, Community health, Patient education, Cancer prevention

## Abstract

Urban–rural disparities in the uptake rate of cervical cancer screening are significant, while one major barrier to rural populations completing the screening is a lack of knowledge. Therefore, implementing health education targeted towards rural populations is crucial. This study aimed to investigate the impact of a theory-driven, culture-tailored educational program on promoting cervical cancer screening among rural Chinese women. The study, a two-arm parallel, non-randomized controlled trial, was conducted in 10 villages. A total of 362 rural women aged 25–64 years (Mean = 45.18, SD = 9.11) were recruited and assigned to the intervention arm or the control arm, with 181 participants in each. Both groups received routine local health education on cervical cancer screening, while the intervention arm also participated in a five-session nurse-led educational program based on social cognitive theory and adapted to the rural Chinese sociocultural context. The primary outcome measured was the cervical cancer screening uptake rate, with secondary outcomes including self-efficacy and knowledge of cervical cancer screening. Data analysis was performed using Chi-square and generalized estimating equation (GEE) models. Results indicated that the intervention arm demonstrated significantly greater improvements in self-efficacy and knowledge than the control arm immediately after the intervention and at three months post-intervention (*p* < 0.001). At six months post-intervention, the screening uptake rate in the intervention arm was significantly higher (*p* < 0.001). Furthermore, 18 months post-intervention, the self-efficacy and knowledge of the intervention arm remained at relatively high levels (*p* < 0.001). The study findings demonstrated that the educational program had a positive impact on increasing participation in cervical cancer screening within rural communities. As a result, the theory-driven and culture-tailored educational program could be incorporated into cancer prevention promotion strategies in rural areas. However, further high-quality randomized control trials are necessary to assess and generalize this educational approach more widely.

*Trial registration* Chinese Clinical Trial Registry, ChiCTR2200055954. Registered 29 January 2022, https://www.chictr.org.cn/showprojEN.html?proj=150955.

## Introduction

Cervical cancer is one of the most frequently diagnosed gynecological cancers^1^ and thus poses a significant threat to women’s health. To promote the prevention and control of cervical cancer, both the American Cancer Society^2^ and the Chinese Centre for Disease Control and Prevention^3^ recommend that sexually experienced women undergo regular cervical cancer screenings from the age of 25. However, in various countries, there are significant disparities between the rate of uptake of cervical cancer screening by women living in urban areas and those living in rural areas^4–6^. The determinants of these disparities have been identified as factors such as health literacy levels, access to healthcare services, and health-related resource distribution^7–9^. Therefore, to improve rural women’s access to cervical cancer screening resources, many countries have implemented national screening programs to provide screening opportunities specifically targeted to women in rural areas^10–13^. In 2009, the Chinese government initiated the National Cervical Cancer Screening Program in Rural Areas (NCCSPRA), an annual public health program that offers free cervical cancer screening services to rural women aged 35–64^14,15^. However, despite the implementation of the NCCSPRA, the overall rate of uptake of cervical cancer screening in rural China remains suboptimal. A population-based study reported that in 2018 and 2019 in China, 41.1% of urban women but only 32.4% of rural women aged 35–64 had undergone screening (*p* < 0.001)^15^. One major factor leading to this suboptimal rate of screening uptake among rural Chinese women is a lack of knowledge of cervical cancer prevention^16,17^. Thus, to eliminate urban–rural disparities, there is a need for health education programs aimed at promoting cervical cancer screening among rural Chinese women.

To the best of our knowledge, there is currently no relevant health education specifically targeted to rural Chinese women. Accordingly, in this study, we developed an educational program targeted at rural Chinese women. This program was based on social cognitive theory, which is widely used in developing health promotion and disease prevention strategies^18^. Previous research has highlighted the importance of tailoring interventions to rural populations and ensuring cultural appropriateness (e.g., using local languages and/or considering the local sociocultural barriers to screening when develop intervention materials ), which could significantly enhance the screening uptake rate of rural populations^19,20^. Additionally, community engagement has been recommended as a facilitator of cervical cancer screening^21–23^. Therefore, the content of our educational program was based on a culturally tailored format, and the program was implemented in a community-based setting. Moreover, prior to the current study, a pilot study was conducted to assess the feasibility of this program in rural Chinese contexts. The results indicated that participating in the educational program led to improvements in rural women’s self-efficacy as well as knowledge of cervical cancer screening^24^.

To further investigate the ability of this theory-driven and culture-tailored educational program to improve cervical cancer screening behavior in rural Chinese populations, the current full-scale study was designed and implemented. We hypothesized that compared with routine health education, the educational program would lead to greater enhancement in rural Chinese women’s self-efficacy and knowledge of cervical cancer screening and their participation in cervical cancer screening.

## Methods

### Study design

The study was conducted in rural villages across two provinces of eastern China using a two-arm parallel, non-randomized controlled pre-and post-test trial design. Due to concerns about potential contamination and the nature of the intervention, randomization at the individual level and blinding were not possible. The report of the study followed the Transparent Reporting of Evaluations with Nonrandomized Designs (TREND) guideline, which was developed to improve the transparency and quality of reporting behavioral and public health evaluations with non-randomized designs^25^. This study was registered on the Chinese Clinical Trial Registry with the trail number ChiCTR2200055954 (29/1/2022).

### Study sites and participants

This study was conducted in rural villages from two provinces (Shandong and Jiangsu) in mainland China. These villages have similar geographic and sociocultural environments and economic levels. Eligible rural women from these villages were recruited as participants.

G*Power version 3.1 (http://www.macupdate.com/apps) was utilized to estimate the sample size. The effect size on cervical cancer screening uptake rate, as reported by previous studies that have implemented health education^26–28^, was taken into consideration. Additionally, an attrition rate of up to 20% ^29^ was considered. Based on these factors, it was determined that a minimum sample size of 354 (177 per study arm) was required for this study.

Convenience sampling was adopted. In China, sexually experienced women are recommended to undergo regular cervical cancer screenings from the age of 25^3^. Furthermore, a three-year interval for cervical cancer screening is suggested^3^. Therefore, in this study, rural women aged 25 and over who had never undergone cervical cancer screening before or had not undergone cervical cancer screening within the previous three years were our target. Initially, we selected and invited villages with similar sociodemographic characteristics but located far apart from each other as our study sites. Subsequently, villages that agreed to participate in this study were assigned to the intervention or control arm in a 1:1 ratio. Next, we collaborated with local healthcare professionals in each village to reach and invite eligible rural women based on the inclusion and exclusion criteria. We also displayed posters in public areas of the villages to encourage eligible rural women to participate in the study voluntarily. The inclusion criteria were (i) aged 25–64, (ii) residing in a rural area with a permanent registered rural address, (iii) a history of sexual activity, and (iv) no current or previous diagnosis of cervical cancer or history of hysterectomy. The exclusion criteria were (i) being pregnant, (ii) having a medical diagnosis of any serious physical illness or mental or cognitive disorder, (iii) having difficulty with oral expression or communication, and (iv) simultaneously participating in other cervical cancer screening-related research.

### Intervention and control strategies

**Fig. 1:**
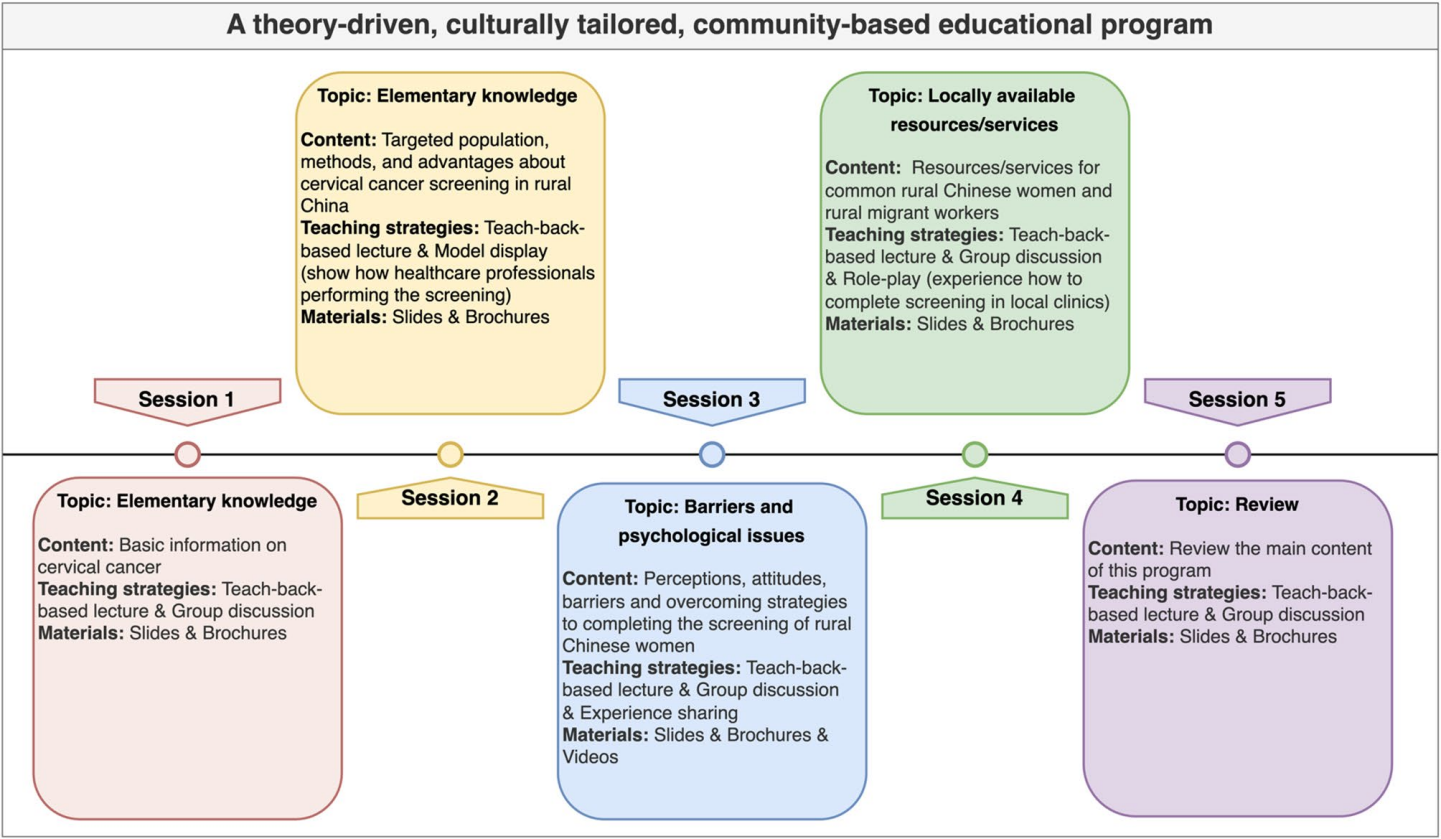
Outline of the five sessions in the educational program.

All participants were exposed to routine health education on cervical cancer screening provided by local healthcare facilities. In addition, the participants in the intervention arm received a theory-driven and culture-tailored educational program. This program consisted of five educational sessions (each session 40–60 min) conducted on a weekly basis (Fig. 1).

#### The theoretical framework of the intervention

Social cognitive theory^18,30^ was applied to guide the development of this educational program. This program aimed to provide the participants with knowledge of cervical cancer screening and to enhance their self-efficacy in screening. This was expected to help them understand the beneficial outcomes of cervical cancer screening and become familiar with the local resources and services of the screening and strategies to overcome the barriers to undergoing screening. Therefore, they would be able to establish a health goal of undergoing screening and ultimately do so.

#### Evidence-based and culture-tailored educational sessions of the intervention

The topics covered in the educational sessions were evidence-based and mainly focused on elementary knowledge of cervical cancer, common barriers to cervical cancer screening completion, specific psychological problems that hinder screening, and locally available screening-related resources and services^20^.

Culture-tailored approach was applied in the program development. Influenced by the collectivism culture of being in harmony with and loyal to the group^31–33^, rural Chinese women were more likely to undergo cervical cancer screening if their family and friends also accept the screening^33^. Therefore, group-based activities were designed in sessions of the program. Confucianism culture^34–36^, which emphasized being introverted and conservative, led to rural Chinese women being introverted and conservative about sex-related topics^34,35^, and open discussions on these were uncommon and even considered as taboo^36^. To break the silence and eliminate the stigma of cervical cancer and receiving relevant examinations, open discussions were arranged in the program. Meanwhile, targeted at the sociocultural issues relevant content was developed in the program, mainly focused on introducing the national cervical cancer screening program, and the specific medical services for migrant workers (rural people coming from rural areas but moving to and temporarily living in large urban cities to seek employment^37^).

#### Delivery mode of the intervention

All five educational sessions were delivered in a nurse-led, face-to-face, group-based format. The available room in the community center of each village was borrowed as the classroom. The group size for each session was limited to 15 participants, and each session ranged from 40 to 60 min. Various teaching strategies were employed during the sessions, such as teach-back-based lectures^38,39^, group discussions, simulation-based learning (including model display and role-play)^40–42^, and experience sharing. Self-developed slides, videos, and brochures were displayed or distributed to the participants during sessions.

### Outcome data collection

E-version of questionnaire was adopted for data collection, using the platform WenJuanXing (https://www.wjx.cn).

#### Baseline data

At baseline (T0), sociodemographic information, cervical cancer screening-related history, and intention to undergo screening were collected from all participants using a self-developed questionnaire.

#### Primary outcome: cervical cancer screening uptake rate

All participants who completed cervical cancer screening post-intervention were required to make a self-report. Meanwhile, we confirmed the screening behavior of all participants by the official medical records with the assistance of local healthcare professionals. At 6 months post-intervention (T3), the screening uptake rate of the intervention and control arms was evaluated and compared.

#### Secondary outcomes: self-efficacy and knowledge

The self-efficacy in cervical cancer screening was assessed using the Cervical Cancer Screening Self-Efficacy Scale (Chinese version), which has a Cronbach’s α of 0.934^43^. This 5-point Likert scale consists of 16 items, with a low score indicating a high level of self-efficacy. The knowledge level of cervical cancer screening was assessed through a self-developed questionnaire consisting of 10 multiple-choice questions. Each correct response to each question gives one point, and the total score of this questionnaire ranges from 0 to 10. A higher score indicates a higher level of knowledge. An expert panel of healthcare professionals reviewed and evaluated this questionnaire and determined that its content validity index was 1.000. At T0, immediately after the intervention (T1), and at 3 months post-intervention (T2), two secondary outcomes, self-efficacy and knowledge were assessed. Furthermore, to evaluate the long-term impact of the program, these two outcomes were measured at 18 months post-intervention (T4) as well.

### Statistical analysis

The intention-to-treat (ITT) principle was adopted for the data analysis. Data analysis was conducted using IBM SPSS version 26.0 (IBM Corp. Armonk, NY). Descriptive statistics were used to summarize the baseline data. Chi-square (χ^2^) and independent t-tests were used to compare the participants’ characteristics between the study arms at baseline. The characteristics with a p-value of less than 0.20 were considered as potential confounding covariates^44^ and were adjusted in the subsequent outcome analysis between the two study arms.

The intention-to-treat principle was applied for outcome analysis to explore the effects of the intervention on the outcomes. A χ^2^ test was used to compare the cervical cancer screening uptake rates at T3 between the two study arms, and a logistic regression model was used to explore the difference with adjustment for the potential confounding covariates. Generalized estimating equation (GEE) models were used to compare the two study arms in terms of changes in the participants’ cervical cancer screening self-efficacy and knowledge levels at T1, T2, and T4 with respect to T0 while adjusting for potential confounding covariates. All statistical tests were two-sided with a significance level of 0.05.

### Consent to participate and ethics approval

Written informed consent was obtained from all participants after they were informed about the study design and agreed to participate. The study was conducted in accordance with the Declaration of Helsinki. The protocol of this study was reviewed and approved by the Joint Chinese University of Hong Kong–New Territories East Cluster Clinical Research Ethics Committee (No. 2021.618).

## Results

**Fig. 2:**
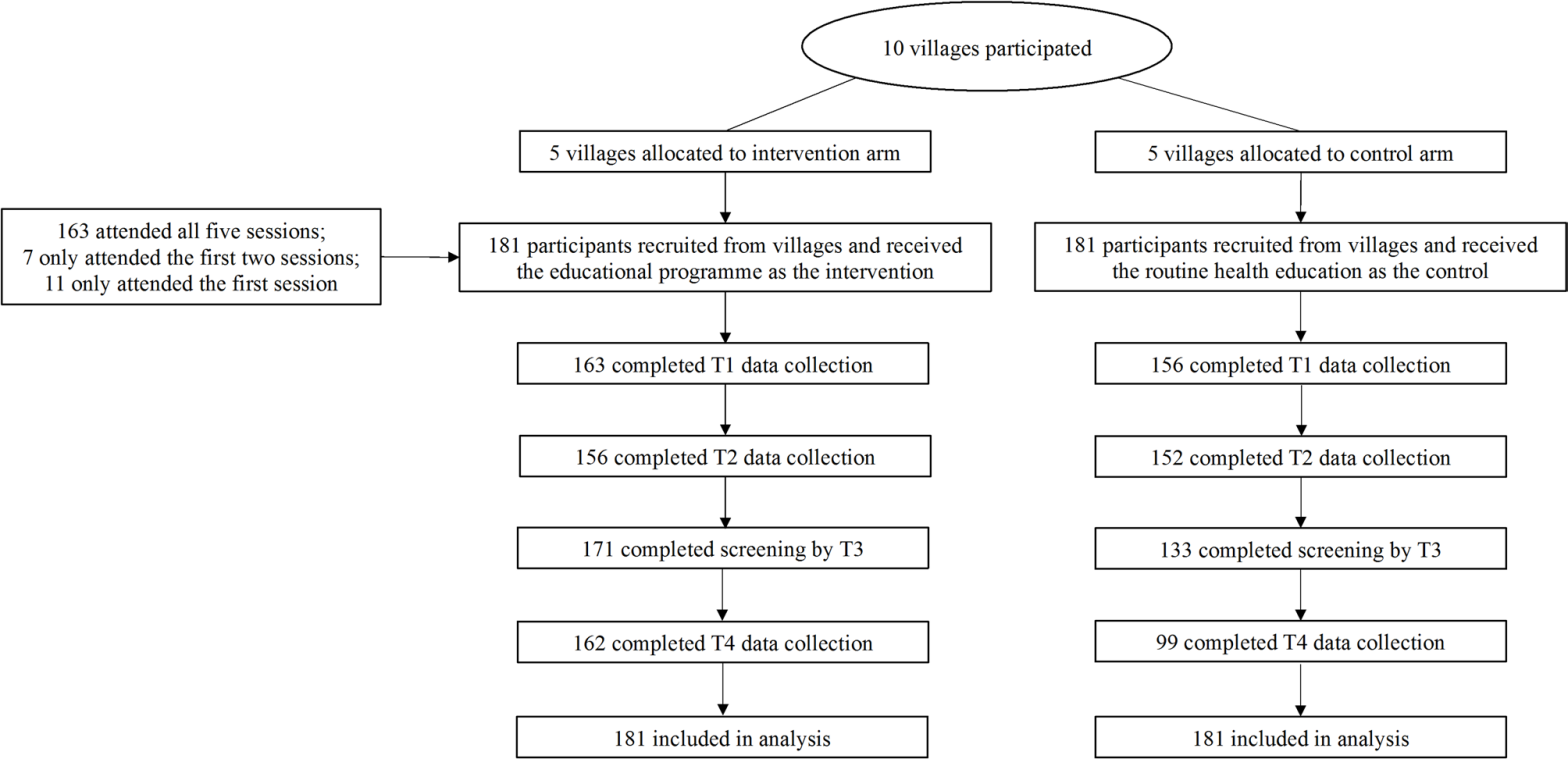
Flow diagram of the recruitment and retention of participants.

Participant recruitment was performed from February to April 2022. A total of 10 villages enrolled in this study, with five villages allocated to the intervention arm (labeled as Village I1, Village I2, Village I3, Village I4, and Village I5) and the other five to the control arm (labeled as Village C1, Village C2, Village C3, Village C4, and Village C5). We recruited 181 participants in each study arm (Table 1), resulting in a total of 362 rural women with an average age of 45.18 (± 9.11) years who participated in this study (Fig. 2). The baseline sociodemographic and cervical cancer screening-related information of all participants is presented in Table 2. Based on the between-arm comparison of baseline data, two variables (number of children and type of employment) were identified as potential confounding covariates (*p* < 0.20).

**Table 1:** Participants recruitment (*N* = 362).

Study arm	Numbers of participants recruited
Intervention arm	
Village I1	47
Village I2	44
Village I3	33
Village I4	31
Village I5	26
Control arm	
Village C1	46
Village C2	51
Village C3	34
Village C4	21
Village C5	29

**Table 2:** Comparison of the baseline information between intervention and control groups (*N* = 362).

Variables	Mean ± SD ^#^ (Range)/*N* (%)			
	Total (*N* = 362)	Intervention group (*n* = 181)	Control group (*n* = 181)	*P* value
Age	45.18 ± 9.11 ( 25–64)	45.30 ± 9.41 (25–63)	45.07 ± 8.82 (25–64)	0.809
Marital status				
Single/Divorce/Widowed	40 (11.0)	21 (11.6)	19 (10.5)	0.737
Married	322 (89.0)	160 (88.4)	162 (89.5)	
Number of children				
0	36 (9.9)	16 (8.8)	20 (11.0)	0.185
1	145 (40.1)	81 (44.8)	64 (35.4)	
≥2	181 (50.0)	84 (46.4)	97 (53.6)	
Duration of settled in this village				
≤ 15	128 (35.4)	60 (33.1)	68 (37.6)	0.379
≥ 16	234 (64.6)	121 (66.9)	113 (62.4)	
Educational level				
Junior high school or below	119 (32.9)	62 (34.2)	57 (31.5)	0.701
Senior high school/Junior college education	170 (47.0)	81 (44.8)	89 (49.2)	
University/college/even higher	73 (20.2)	38 (21.0)	35 (19.3)	
Type of employment				
Unemployed/Self-employed	119 (32.9)	52 (28.7)	67 (37.0)	0.093
Fixed occupations	243 (67.1)	129 (71.3)	114 (63.0)	
Monthly income per capita (5000 CNY ≈ 700USD)				
≤ 5000 CNY	197 (54.4)	95 (52.2)	102 (56.4)	0.460
> 5000 CNY	165 (45.6)	86 (47.5)	79 (43.6)	
Have a medical insurance				
Yes	349 (96.4)	176 (97.2)	173 (95.6)	0.397
No	13 (3.6)	5 (2.8)	8 (4.4)	
Have religious belief				
Yes	38 (10.5)	17 (9.4)	21 (11.6)	0.493
No	324 (89.5)	164 (90.6)	160 (88.4)	
Previous cervical cancer screening				
Never had screening before	211 (58.3)	107 (59.1)	104 (57.5)	0.749
≥ One time, 3 years ago or more	151 (41.7)	74 (40.9)	77 (42.5)	
Intention to undergo cervical cancer screening				
Yes, willing to	151 (41.7)	75 (41.4)	76 (42.0)	0.927
Not sure	167 (46.1)	85 (47.0)	82 (45.3)	
No, not willing to	44 (12.2)	21 (11.6)	23 (12.7)	

After finalizing participant recruitment, we conducted a series of five educational sessions for participants from the intervention arm villages—Village I1, I2, I3, I4, and I5—over five weekends from April to May 2022. In line with our intervention protocol, which limited the number of participants to 15 per session, we organized the participants into smaller subgroups: Village I1 and I2 each had four subgroups, Village I3 and I4 each had three subgroups, and Village I5 had two subgroups. Each weekend, we held separate sessions for each subgroup, ensuring that all subgroups across the villages of the intervention arm received the same educational content within the same week.

Specifically, on each Saturday, we delivered the same educational session in the morning to all four subgroups from Village I1 and in the afternoon to all four subgroups from Village I2. On each Sunday, we provided the same session in the morning to all three subgroups from Village I3, in the early afternoon to all two subgroups from Village I5, and in the late afternoon to all three subgroups from Village I4. This approach ensured that each subgroup could receive the educational content exactly once per session, and with five sessions conducted over five weeks, each subgroup could participate in all five sessions.

The results showed that of all 181 participants in the intervention arm, 163 participants (90.1%) attended all five sessions of this program. This study was conducted during the coronavirus disease 2019 (COVID-19) pandemic and the main reason for participants not completing all sessions was related to the pandemic response policies, such as quarantine measures.

### Impact on cervical cancer screening uptake rate

By T3, of 181 participants in the intervention arm, 171 completed cervical cancer screening, while 133 of 181 participants in the control arm completed it. The screening uptake rate in the intervention arm was significantly greater than that in the control arm (94.5% vs. 73.5%; χ^2^ = 29.647; odds ratio (OR) = 6.171; 95% confidence interval (CI): 3.010, 12.653; *p* < 0.001). Logistic regression was used with adjustment for the abovementioned potential confounding covariates, and the result showed the difference in uptake rate between the two arms remained significant (adjusted OR = 6.195; 95% CI: 3.019, 12.713; *p* < 0.001).

### Impact on self-efficacy and knowledge of cervical cancer screening

For the missing data on self-efficacy and knowledge due to the participants’ loss to follow-up at T1, T2, and T4, multiple imputation was adopted (Table 3). GEE models were applied to explore the program’s effects on self-efficacy and knowledge with adjustment for the potential confounding covariates. The results of the GEE analysis are presented in Table 4. By comparing the changes in self-efficacy between the two study arms, we found that the intervention arm showed a significantly greater increase in the level of self-efficacy than the control arm at T1 (β= − 16.753; 95% CI: − 19.515, − 13.992; *p* < 0.001), T2 (β = − 10.297; 95% CI: − 13.149, − 7.446; *p* < 0.001), and T4 (β = − 23.184; 95% CI: − 25.921, − 20.448; *p* < 0.001). Regarding the changes in knowledge, the intervention arm showed a significantly greater increase in the knowledge level than the control arm at T1 (β = 3.066; 95% CI: 2.545, 3.586; *p* < 0.001), T2 (β = 1.216; 95% CI: 0.752, 1.680; *p* < 0.001), and T4 (β = 1.277; 95% CI: 0.753, 1.801; *p* < 0.001).

**Table 3:** The changes in participants’ self-efficacy and knowledge among over time.

a. Summary of the variables’ changes over time (with missing data)				
Variable	Mean ± SD			
	T0 (*N* = 362)	T1 (*N* = 319)	T2 (*N* = 308)	T4 (*N* = 261)
Self-efficacy				
Intervention arm	50.63 ± 12.45 (*n* = 181)	29.74 ± 5.84 (*n* = 163)	27.64 ± 5.71 (*n* = 156)	26.52 ± 7.47 (*n* = 162)
Control arm	49.88 ± 11.54 (*n* = 181)	47.28 ± 6.91 (*n* = 156)	37.43 ± 9.61 (*n* = 152)	48.12 ± 5.88 (*n* = 99)
Knowledge				
Intervention arm	4.38 ± 1.93 (*n* = 181)	7.26 ± 0.96 (*n* = 163)	6.97 ± 0.53 (*n* = 156)	6.85 ± 1.28 (*n* = 162)
Control arm	4.42 ± 2.07 (*n* = 181)	4.11 ± 2.17 (*n* = 156)	5.64 ± 1.65 (*n* = 152)	5.40 ± 1.73 (*n* = 99)

b. Summary of the variables’ changes over time (with multiple imputation)				
Variable	Mean ± SD ^#^			
	T0	T1	T2	T4
Self-efficacy				
Intervention arm (*n* = 181)	50.63 ± 12.45	30.34 ± 5.96	27.53 ± 6.07	26.57 ± 7.32
Control arm (*n* = 181)	49.88 ± 11.54	46.35 ± 7.41	37.08 ± 9.69	49.01 ± 5.89
Knowledge				
Intervention arm (*n* = 181)	4.38 ± 1.93	7.25 ± 0.98	6.91 ± 0.66	6.84 ± 1.31
Control arm (*n* = 181)	4.42 ± 2.07	4.23 ± 2.18	5.74 ± 1.56	5.61 ± 1.74

**Table 4:** GEE model for comparing the self-efficacy and knowledge between intervention and control arms (*N* = 362).

Parameter	β (95% CI)	*P* value
Self-efficacy		
Group ^a^	0.814 (− 1.587, 3.215)	0.507
T1 ^b^	− 3.532 (− 5.408, − 1.656)	< 0.001
T2 ^c^	− 12.802 (− 14.887, − 10.717)	< 0.001
T4 ^d^	− 0.872 (− 2.686, 0.941)	0.346
Group*T1 ^e^	− 16.753 (− 19.515, − 13.992)	< 0.001
Group*T2 ^f^	− 10.297 (− 13.149, − 7.446)	< 0.001
Group*T4 ^g^	− 23.184 (− 25.921, − 20.448)	< 0.001
Knowledge		
Group ^a^	− 0.061 (− 0.455, 0.334)	0.763
T1 ^b^	− 0.193 (− 0.611, 0.225)	0.365
T2 ^c^	1.317 (0.965, 1.670)	< 0.001
T4 ^d^	1.188 (0.788, 1.588)	< 0.001
Group*T1 ^e^	3.066 (2.545, 3.586)	< 0.001
Group*T2 ^f^	1.216 (0.752, 1.680)	< 0.001
Group*T4 ^g^	1.277 (0.753, 1.801)	< 0.001

Covariates adjusted: number of children, type of employment.

^a^ Group = Between-group difference of intervention and control groups at baseline.

^b^ T1 = Change in the control group at T1 compared with T0.

^c^ T2 = Change in the control group at T2 compared with T0.

^d^ T4 = Change in the control group at T4 compared with T0.

^e^ Group*T1 = Compared with control group, additional change in the intervention group at T1.

^f^ Group*T2 = Compared with control group, additional change in the intervention group at T2.

^g^ Group*T4 = Compared with control group, additional change in the intervention group at T4.

## Discussion

In this non-randomized controlled trial, we implemented a theory-driven, culturally tailored, and community-based educational program in rural China. The results showed that the program enhanced the cervical cancer screening attitudes, leading to an increase in the screening uptake rate among rural Chinese women.

The results of post-intervention follow-up showed that the cervical cancer screening self-efficacy and knowledge levels of the intervention arm were significantly higher than that of the control arm. The impact of our educational program on improving rural women’s knowledge of cervical cancer screening aligned with the findings from previous studies^45,46^. However, the influence of the educational program on enhancing the self-efficacy level was inconsistent with the results in previous studies,^26,46^ which found that social cognitive theory-based educational interventions in rural women led to no significant changes in their level of self-efficacy in screening. This may be because unlike the intervention designs of the above-mentioned studies, which used lay health workers for health education,^26,46^ our educational intervention was led by a healthcare professional, a Registered Nurse. The professional’s involvement might contribute to enhancing the intervention intensity and positive effects. Additionally, our educational program employed various teaching strategies which were reported effective in enhancing self-efficacy in previous studies^38–41^. Thus, in this study, participants’ self-efficacy in cervical cancer screening could be enhanced.

Besides the increases in the level of self-efficacy in cervical cancer screening and level of knowledge, the screening uptake of intervention group was also improved. At T3, cervical cancer screening uptake rate of the intervention arm reached 94.5%, which was significantly larger than the control group (*p* < 0.001). This significant improvement in post-intervention screening uptake rate in the intervention arm aligned with the findings of previous studies^26,28^. The remarkable progress in the screening uptake rate may be attributable to the theory-driven design of our educational program. Our program was developed based on social cognitive theory, a well-structured behavioral change-related theory that has been widely and effectively applied in improving cancer-related behavioral changes^47^. Moreover, our program was developed in a culturally appropriate format. Studies have emphasized the importance of applying culturally appropriate interventions for cancer control in rural populations^21,22^. Therefore, by targeting rural Chinese women and tailoring the intervention to specific cultural elements, our program was well-suited to rural Chinese contexts and may be significantly effective in improving relevant outcomes.

Additionally, we evaluated the long-term impact of the educational program on participants’ self-efficacy and knowledge of cervical cancer screening. We found that at T4, although most participants had already completed the screening, the intervention arm could still maintain relatively higher levels of self-efficacy and knowledge. The social cognitive theory illustrated sufficient self-efficacy and knowledge could effectively promote behavioral change^18^. Therefore, maintaining high levels of self-efficacy and knowledge might make the participants well-prepared for completing their next cervical cancer screening behavior, even forming the habit of regularly undergoing screening in the future.

We also observed that the control arm’s self-efficacy and knowledge levels regarding screening and rate of uptake of screening showed some positive changes over time. Noteworthily, during the post-intervention follow-up period, the annual national screening program, the NCCSPRA^14^, was started. Besides the routine health education of cervical cancer screening provided by local healthcare facilities, all participants were exposed to the promotion of the national program as well, which had a beneficial impact on their self-efficacy and knowledge levels, and screening uptake rates. Hence, these outcomes in the control arm also improved. However, a comparison of the results of the intervention and control arms revealed that the positive effects of routine public health education and promotion on rural women’s self-efficacy and knowledge of cervical cancer screening were limited. The screening uptake rate in the control arm was still lower than that in the intervention arm. Therefore, in the future, healthcare providers could consider incorporating our educational program into their routine health education practice and the promotion activities of the national screening program, to further promote cervical cancer screening among the rural populations.

## Strengths and limitations

This study has several strengths. First, the participants were recruited from multiple villages in two provinces. This enhanced the diversity of participants and the generalizability and impact of the results. Second, we conducted long-term follow-ups to observe the impact of our educational program. This was important, as cervical cancer screening is not a one-time test. Thus, by assessing the long-term impact of our program on participants’ level of self-efficacy in screening and level of knowledge of screening, we could explore whether it encouraged them to consistently seek screening in the future. Moreover, to the best of our knowledge, the program is the first evidence-based and culturally tailored educational program on cervical cancer screening targeting rural Chinese women. The implementation of this program contributed to an increase in the rate of uptake of screening among the intervention arm, thereby demonstrating that the program reduces the urban–rural disparities in cervical cancer control and enhances health equity.

Nevertheless, this study had some limitations. First, a major limitation was the non-randomized design. This design was used because it is considered inappropriate to conduct an individual-level randomized controlled trial in a community-based study, given the increased risk of contamination due to only half of the individuals of the same community receiving an intervention^48^. Therefore, in the current study, the group allocation was conducted at the community level, and a quasi-experimental design was applied. In future research and if resources permit, the use of a cluster-randomized controlled trial design could be considered. Moreover, convenience sampling was adopted in this study which might lead to selection bias and threaten the internal validity of the study findings. To reduce the bias, we recruited rural women from multiple villages with different modes to enhance the diversity and variety of participants and the representativeness of the study sample. Second, self-reported scales and questionnaires were used for evaluating outcomes. This introduced the possibility of bias due to participants inaccurately assessing their own health status. To mitigate this bias, we provided clear instructions to the participants on how to accurately evaluate their situations and complete the questionnaires. We also emphasized the anonymity of the study to build trust and encourage the participants to report their actual status.

## Conclusion

Promoting cervical cancer screening among rural populations is crucial to cervical cancer control. The findings of this study demonstrate that the implementation of our social cognitive theory-driven, culturally tailored, community-based educational program positively impacted the screening self-efficacy and knowledge levels of a sample of rural women in China and increased the screening uptake rate. Considering the program’s culturally tailored design, when generalizing this program to rural health practices in different contexts, it may be necessary to modify the intervention components and content to suit the sociocultural settings of given contexts. Furthermore, future research using a pragmatic cluster-randomized controlled trial design is needed to evaluate the effectiveness of health education interventions in promoting cervical cancer screening among rural populations.

## Data Availability

The data analyzed during the current study are available by contacting the corresponding author upon reasonable request.
